# Effects of landscape structure and patch characteristics on the density of central populations of the eastern green lizard *Lacerta viridis*

**DOI:** 10.1002/ece3.10419

**Published:** 2023-08-17

**Authors:** Ana María Prieto‐Ramírez

**Affiliations:** ^1^ Institute of Geography University of Hildesheim Hildesheim Germany

**Keywords:** habitat loss, landscape structure, lizards, population density, scale of effect

## Abstract

A better understanding of the impact of habitat loss on population density can be achieved by evaluating effects of both parameters within remnant habitat patches and parameters of the landscape surrounding those patches. The integration of predictors at the patch and landscape level is scarce in animal ecological studies, especially for reptiles. In this study, a patch–landscape approach was applied to evaluate the combined effects of within‐patch habitat quality, patch geometry and landscape configuration and composition on the density of remnant populations of the eastern green lizard, *Lacerta viridis*, in a highly modified landscape in Bulgaria. Landscape composition variables (proportion of different land covers) were measured at different spatial scales surrounding patches. Single‐scale models were built to evaluate combined effects of all predictors on density, when including all landscape composition variables at a specific spatial scale. Multi‐scale models were applied to analyze combined effects when including landscape composition variables at the scale of their strongest effect (scale of effect, SoE). Results showed that the SoE of proportion of cropland and urban areas was small (50 m), while for proportion of habitat was large (1.5 km). The overall effect of habitat loss was better explained by the multi‐scale model. Population density increased with patch area and decreased with patch shape irregularity and with the proportion of three land cover types surrounding patches—cropland, urban areas, and habitat. Combining patch and landscape parameters is important to identify ecological processes that occur simultaneously at different spatial levels and landscape scales, which would imply the application of multi‐scale approaches for the protection of wild animal populations. Results are contrasted with what is known about occupancy patterns of the species in the same region and approaches to integrate both occupancy and density, in the field design of animal ecological studies are suggested.

## Graphical abstract

In this study, combined effects of within‐patch habitat quality, patch geometry, and landscape structure on the density of remnant populations of the eastern green lizard Lacerta viridis, were evaluated at different spatial scales. The overall effect of habitat loss was better explained by the multi‐scale model. Population density increased with patch area and decreased with patch shape irregularity, and with the proportion of three land cover types surrounding patches—cropland, urban areas, and habitat. Combining patch and landscape parameters is important to identify ecological processes that occur simultaneously at different spatial levels and landscape scales, which would imply the application of multi‐scale approaches for the protection of wild animal populations.

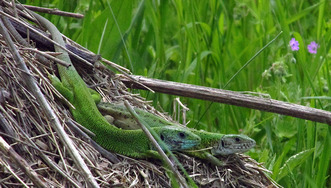

## 1 INTRODUCTION

Reduced population density and abundance are among the main negative effects of habitat loss on wild animal populations and can lead to the extirpation of local populations and changes in the distribution of species (Bender et al., 1998; Tischendorf et al., 2005). Most knowledge about these negative effects and the ecological processes that they trigger resulted from research on birds and mammals (e.g., Bender et al., 1998; Thornton et al., 2011). However, comparatively lower vagility and higher sensitivity to environmental changes make reptiles more vulnerable to negative effects of landscape modification (Doherty et al., 2020).

The most tested parameters in studies of population density and abundance of reptile species are patch area, isolation, and landscape type. Effects of patch area and isolation are highly species‐ and landscape‐dependent. In the case of patch area, several multi‐species studies found positive effects on the abundance of some species and no effect on others (Carvajal‐Cogollo & Urbina‐Cardona, 2008; Delaney et al., 2021; Rizkalla & Swihart, 2006; Shirk et al., 2014), and some authors have reported negative effects (Lion et al., 2016). Such contrasting effects fit meta‐analysis findings of Bender et al. (1998) about patch size effects on density and abundance being negative for edge species, positive for interior species, and negligible for species using both patch edge and interior. Effects of isolation have also been found to be either negative (Carvalho Jr. et al., 2008; Sato et al., 2014; Williams et al., 2012), positive (Lion et al., 2016), or non‐existent (Delaney et al., 2021; Lizana‐Ciudad et al., 2021) on population abundance of reptile species. Moreover, it is known that isolation effects are dependent of species sensitivity to matrix, which determines immigration and emigration rates affecting density and abundance (Tischendorf et al., 2005).

At the landscape level, most studies testing effects of habitat loss on population density and abundance of reptile species apply categorical approaches to compare between different types of landscapes. Thus, for several species, lower abundance has been linked with fragmented landscapes compared to non‐fragmented ones (de Andrade et al., 2019; Leavitt & Fitzgerald, 2013; Walkup et al., 2017) or with specific management practices compared to absence of management (Barrows & Heacox, 2021; Biaggini & Corti, 2015; Kaunert & Mcbrayer, 2015).

Although approaches applied in those studies have allowed to understand the effects of habitat loss on reptiles, two main knowledge gaps remain: First, how do continuous parameters of landscape configuration and composition around remnant habitat patches affect population density and abundance of reptiles? (but see Rizkalla & Swihart, 2006); and second, how do landscape, patch and within‐patch parameters affect simultaneously population density and abundance? Only few studies have integrated these different spatial levels (Barrows & Heacox, 2021; Carvalho Jr. et al., 2008; Sato et al., 2014). Closing these gaps would allow not only to identify relative effects at different spatial levels (landscape, patch, and within‐patch) but also those of landscape configuration and composition separately, and the spatial scales (sensu Martin & Fahrig, 2012) around focal habitat patches at which their effects are strongest (e.g., Lion et al., 2016). This is especially important in the face of two main theories in landscape ecology, the fragmentation threshold hypothesis (Andrén, 1994) and the habitat amount hypothesis (HAH; Fahrig, 2013), which state that the effects of isolation and patch area are highly dependent on the total amount of habitat in the landscape. Therefore, evaluating different spatial levels and types of predictors can lead to a better understanding of the effects of modified landscapes on reptile species populations.

As for other taxa, despite density and abundance being important population traits to identify possible decline preceding population extirpation, effects of habitat loss on reptiles have been much more investigated through population persistence indicators like occupancy (e.g., Biaggini & Corti, 2021; Paterson et al., 2021; van Heezik & Ludwig, 2012). Occupancy can be a much more cost‐effective parameter in terms of data collection, analysis, and interpretation of species distribution (Casner et al., 2014; Sewell et al., 2012). However, factors ruling extinction‐colonization processes can differ from those defining the demographic processes that underline density and abundance (He & Gaston, 2000; Orrock et al., 2000). Such differences have already been reported in the reptile literature (Driscoll et al., 2012; Hubbard et al., 2016; Lizana‐Ciudad et al., 2021), and in cases in which the same environmental factors affect both occupancy and abundance, the direction of the effect is the opposite (Dibner et al., 2017; Rizkalla & Swihart, 2006). Some authors argue that these differences can be present due to factors influencing occupancy acting at larger scales compared to those affecting abundance and density (He & Gaston, 2000; Wilson et al., 2016).

**FIGURE 1:**
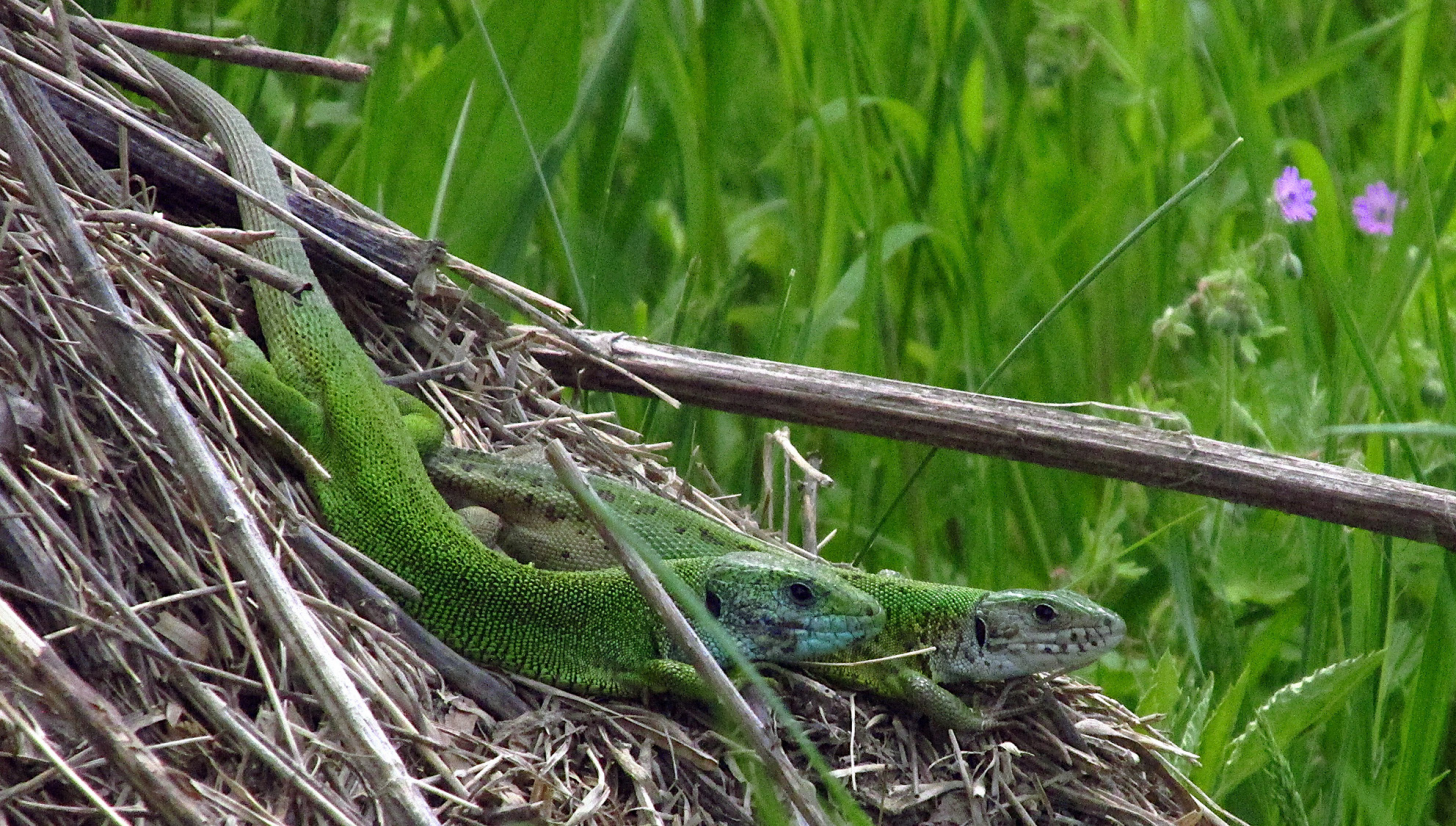
Pair of *Lacerta viridis* (male on the left, female on the right) during the reproduction season.

In this study, I investigated effects of habitat loss on the density of populations of the eastern green lizard *Lacerta viridis* (Figure 1) inhabiting a modified landscape in central Bulgaria. I applied a patch–landscape approach integrating landscape parameters across spatial scales with patch and within‐patch parameters. Effects of habitat loss on occupancy patterns of *L. viridis* have recently been investigated in the same study system (Prieto‐Ramírez et al., 2020), with occupancy being found to be mostly defined by landscape configuration, with the strongest effect of the overall habitat loss process occurring at the 750 m scale around patches. No negative effect of isolation was found, and at the patch level, occupancy depended on patches with both long perimeter and enough core area in the interior, indicating that the species uses not only the border but also the interior of patches. Within‐patch habitat quality was not determinant for occupancy but had positive effect. Based on predictions from literature and findings on the species' occupancy patterns I hypothesize: (1) positive effect of within‐patch habitat quality on population density, (2) no effect of patch area, (3) no effect of isolation, and (4) an effect at small spatial scales of individual landscape composition parameters, as well as of the overall habitat loss process.

## 2 METHODS

### 2.1 Model species and study area

*Lacerta viridis* has a broad distribution range covering Asia Minor, Eastern Europe, and the Balkan Peninsula (Kwet, 2005; Nettmann & Rykena, 1984). Although it is a generalist species that uses a wide range of habitat types, its habitat is fragmented across the whole distribution range (Elbing et al., 1997), and therefore, is protected by the European Habitats Directive (2007) under Annex IV. Moreover, the species is known to have a low dispersal tendency, mainly during natal dispersal and for shorter distances compared to other green lizards (Elbing, 2000; Schneeweiss, 2001), which increases its sensitivity to habitat loss (Chichorro et al., 2019; Henle et al., 2004).

**FIGURE 2:**
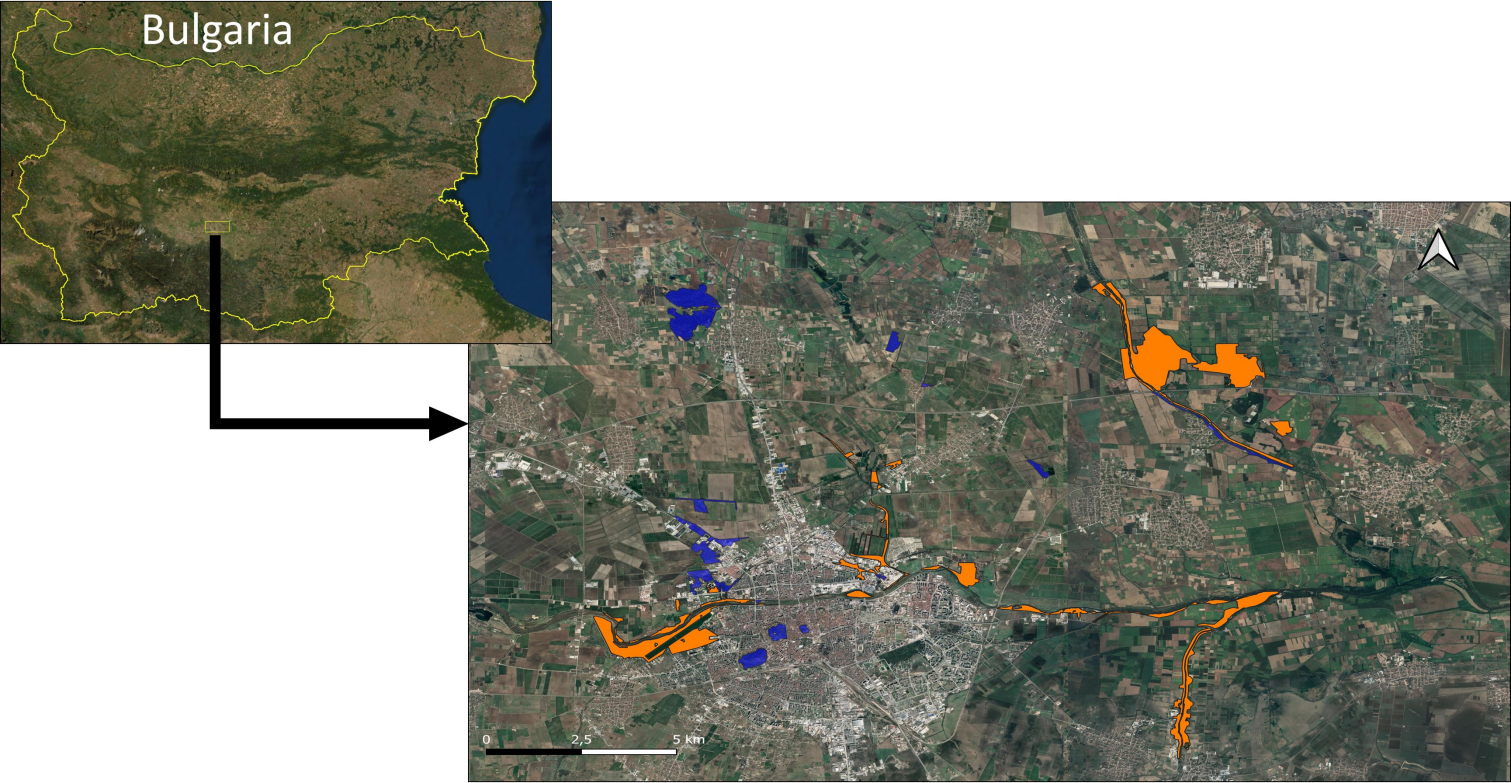
Study site located in the surroundings of Plovdiv, Bulgaria. In color are highlighted the 42 patches surveyed. The species was found to be present in 24 patches (orange) and was not detected in the 18 remaining patches (blue). Only occupied patches were included in the calculation of population density.

The study area was located in the Thracian Plain of Bulgaria, in the surroundings of the city of Plovdiv (Figure 2). This region, which corresponds to part of the current and historical center of the distribution range of the species (Marzahn et al., 2016), is an alluvial plain dominated by the banks of the Maritsa River and its tributary rivers. Here, *L*. *viridis* inhabits diverse natural and semi‐natural habitats, including river banks, shrublands, and mesophilic mixed forest (Mollov, 2011). Urban and agricultural expansion in the region have reduced the habitat of the species (Kambourova‐Ivanova et al., 2012; Mollov & Georgiev, 2015), which is now composed mostly by habitat patches of variable size separated by a matrix of unsuitable land covers.

### 2.2 Survey design

The present study was carried out in the context of a broader study that included collected and analyzed data on occupancy (Prieto‐Ramírez et al., 2020). Therefore, the applied survey design corresponds to a mixed designed suitable for both occupancy and density. Data collection was carried out from beginning of April to late May in 2014. Patches to be visited were selected and identified on satellite imagery available in Google Earth, based on information regarding species requirements in the region and available information on the species distribution. All selected patches are separated from each other by agricultural landscape, urban areas and/or highways. Forty‐two habitat patches were visited in 2014 (Prieto‐Ramírez et al., 2018), from which 24 patches were occupied (Figure 2). Given differences in the factors affecting occupancy and abundance, only data from the 24 occupied patches were used for the present study (Dibner et al., 2017; Fletcher et al., 2005).

Surveys were designed following the protocol proposed by Mackenzie and Royle (2005) for occupancy, prescribing a specific number of visits depending on the probability of detection of the species. Based on estimates of detection probability for similar species (Janssen & Zuiderwijk, 2006; Sewell et al., 2012), the number of surveys per patch was set to two, one in the morning (9:00–12:00 a.m.) and one in the afternoon (14:00–19:00 p.m.) of the same day or 1 day later, in accordance with the species' daily activity pattern (Korsós, 1983). Active surveys lasted 1 h each, walking along predetermined line transects. With a standard walking speed of 20 m/min, which is slow enough to detect lizards, a 1‐h survey corresponds to a total length of 1200 m that were divided into the predetermined transects for each patch. Because most patches had a heterogenous composition, which might imply non‐homogeneous distribution of animals, the number and length of transects was adjusted to represent the different habitat types and the area covered by each into each patch. Nevertheless, all the transects in a patch always summed up 1200 m to assure 1‐h visit. Satellite imagery was used to define the relative coverage of each habitat type within each patch. Transect lengths varied between 50 and 400 m and were randomly located into each within‐patch habitat type, at least 100 m apart from each other. The total length of each transect was placed in only one habitat type. The number of transects surveyed per patch ranged from three to 12. Distance sampling (Buckland et al., 1993) was applied to record the information necessary to calculate density. During transect walking, a width of 2.5 m was scanned at each side of the transect to visually search for *L*. *viridis*, and every time a lizard was detected, the perpendicular distance from the transect to the detection point was measured and recorded.

### 2.3 Calculation of patch variables and landscape structure

**FIGURE 3:**
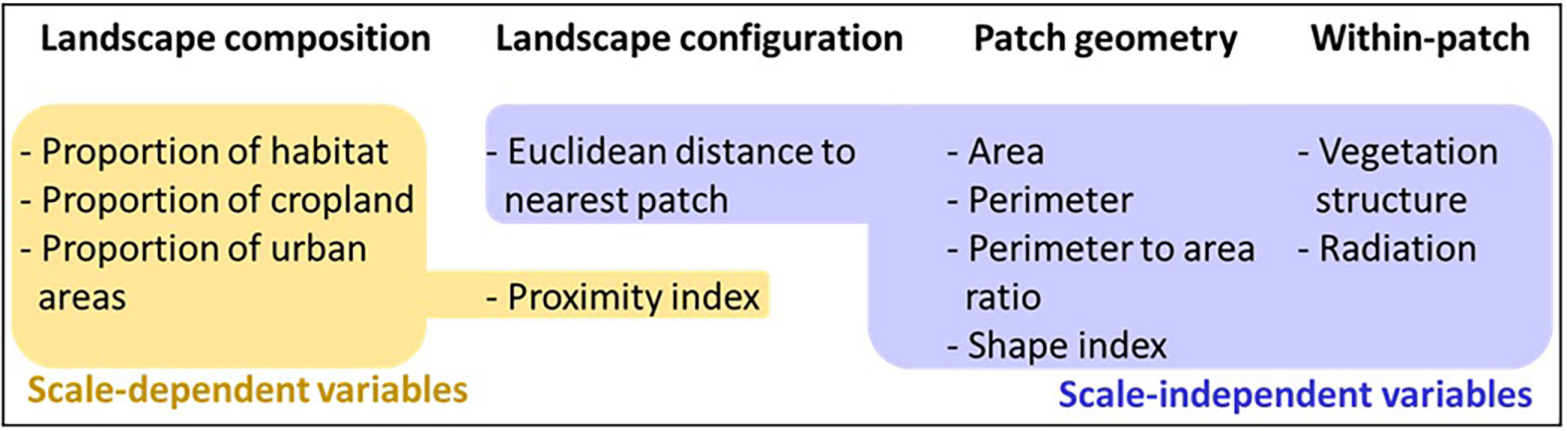
Predictor variables tested. Scale‐dependent variables were measured in all buffers (scales) surrounding single patches.

A patch–landscape approach was applied to analyze the influence of landscape structure and patch characteristics on density. At the landscape level, predictors include variables representative of landscape configuration and landscape composition; at the patch level, variables describe patch geometric characteristics and at within‐patch level, habitat quality variables are included (Figure 3).

Landscape configuration is represented by the distance of each patch to the river (Distance to river) and by two measures of isolation, the edge‐to‐edge Euclidean distance to the nearest patch (np_dist) and proximity index. The proximity index (Gustafson & Parker, 1994), hereafter “prox,” is a scale‐dependent measure of isolation and is calculated as the sum of the ratios patch area/distance to the focal patch for all patches that fall, at least partially, into the buffer of a given distance around the focal patch. Landscape composition variables included the proportion of habitat, cropland, and urban areas surrounding each patch. These variables were calculated using available land cover maps of the region (Prieto‐Ramírez et al., 2020), and were measured at various buffer distances (hereafter, “scales”) around each patch. Scales were selected based on reported dispersal distances for *L*. *viridis* (Grimm et al., 2014; Mangiacotti et al., 2013; Saint‐Girons & Bradshaw, 1989) and include 50, 150, 250, 500, 750 m, 1, 1.5, 2, 2.5, and 3 km. As prox is also a scale‐dependent variable, it was also measured at each scale.

Patch geometry variables included area, perimeter, perimeter to area ratio (Per_area), and shape index (Shape_index). Within‐patch habitat quality was defined based on important parameters found for this species and included vegetation structure and solar radiation (Böker, 1990; Moser, 1998; Prieto‐Ramírez et al., 2018; Waitzmann & Sandmaier, 1990). Vegetation structure was calculated based on available information at the microhabitat level collected at 25 m^2^ plots around several points along transects, as described in Prieto‐Ramírez et al. (2018). Solar radiation was calculated from the digital elevation model, available from the US Geological Survey, with the “Potential incoming solar radiation” module of SAGA (Conrad et al., 2015). Precise description of the calculation of solar radiation can be found in Prieto‐Ramírez et al. (2020). All other calculation procedures were carried out with ArcMap version 10.3.1 (ESRI, 2018), except for shape_index and prox which were calculated with FRAGSTATS version 4 (McGarigal et al., 2012).

### 2.4 Density estimation

As a fixed effort design was applied in the survey, the proportion of area covered by transects was non‐homogeneous across patches. Therefore, estimation was restricted to relative density (density only in the recovered area) instead of abundance. Estimation was done using Distance software (Cassey, 1999; Thomas et al., 2010). First, fitting a detection probability function, and then, applying this function to calculate density in each patch.

Because not all patches had enough data to fit a separate detection function per patch, global detection probability estimation using all data were applied, and afterward, a stratified density estimation was performed. Two types of models were fitted to find the best detection probability model: conventional distance sampling (CDS) model without covariates influencing detection, and multivariate conventional distance sampling (MCDS) with vegetation structure as a covariate determining detection. For both models, all combinations resulting from three functions (uniform key, half‐normal key, hazard rate), three types of adjusted terms (cosine, Hermit polynomial, and simple polynomial) and two methods for calculating encounter rate variance (empirically or assuming distribution of observations as Poisson) were tested. Detection probability model was selected based on Akaike information criterion (AIC), model precision indicated by the coefficient of variation (%CV), and Kolmogorov–Smirnov test (K‐S test) of goodness of fit (See Appendix 1 for values of all tested models). Among the models with DeltaAIC ≤2 and with the highest goodness of Fit (K‐S test: estimate = 0.1199, *p*‐value = .0924), the highest precision was found for all combinations of CDS models with uniform key function (%CV = 4.02). For this set of models, both simple and Hermit polynomial resulted in the same number of adjusted terms (2). Therefore, global detection probability was calculated from a CDS model with uniform key function and hermit polynomial adjusted terms. Although having the same precision, AIC, and goodness of fit, the model with empirical calculation of encounter rate variance was selected over the one with predetermined Poisson distribution, because it calculates variance from the observed data (Buckland et al., 2015).

To estimate density, data from temporal replicates were pooled together in each transect, only data overlapping within a 5 m radius was discarded as it might be the same individual. Detection probability function was applied by adding the estimated global detection probability and standard error as global multipliers. Settings for detection were specified as uniform key function with no adjusted terms for detection not to be computed again. To estimate relative density, area was set to zero and encounter rate settings were defined assuming a Poisson distribution with overdispersion factor set to zero, as applied in other studies on lizard's relative density (de Andrade et al., 2019).

### 2.5 Statistical analysis

To find the relevant scales at which density is explained I tested whether density is explained at single scale(s) or simultaneously at multiple scales. Single‐scale models included all scale‐dependent variables (proportion of habitat, proportion of cropland, proportion of urban areas, and prox) calculated at the same scale, plus non‐scale‐dependent variables (np_dist, patch geometry variables and within‐patch variables). Multi‐scale models included each scale‐dependent variable at its scale of effect (SoE), together with non‐scale‐dependent variables. To identify the SoE of each scale‐dependent variable, univariate models with each of these variables were fitted at each scale. The scale with the highest Nagelkerke *R* ^2^ (N*R* ^2^) was selected as the SoE. In cases when the highest N*R* ^2^ value was present at several scales, the smallest scale was selected.

Data were analyzed applying generalized linear models with Gamma error distribution and “logit” link in the program R (R Core Team, 2022). The following steps were applied to each single‐scale dataset and to the dataset of the multi‐scale approach. To avoid collinearity among variables to be included in the same model, variables correlations were tested by means of Spearman rank correlation test. If two variables were correlated (*r*s > .60), several global models were built up, each of them including only one of the correlated variables. Additionally, the variance inflation factor (vif) was calculated for each global model, and variables with vif < 10 were retained.

The global model (or models, depending on variables' correlations) was tested for spatial autocorrelation of residuals by means of Global Moran's I test. Then, all models with all possible combinations of the variables included in the global model were generated with the *dredge* function of the MuMIN package in R (Barton, 2015). Model selection was performed in two steps: first, based on AICc (DeltaAICc ≤2), and then, based on N*R* ^2^ and on deviance reduction from the null model obtained through a goodness of fit *F*‐test (hereafter “deviance change”). Comparisons across single scales, and of these with multi‐scale models were done based on N*R* ^2^ and deviance change.

## 3 RESULTS

Density estimation of the 24 populations studied ranged between 115.31 and 1953.5 individuals/km^2^, with a mean of 536.7 individuals/km^2^ (see Appendix 2 for complete data on population's density estimates and their specific location). No spatial autocorrelation for residuals was found in any global model.

### 3.1 Scale of effect

**FIGURE 4:**
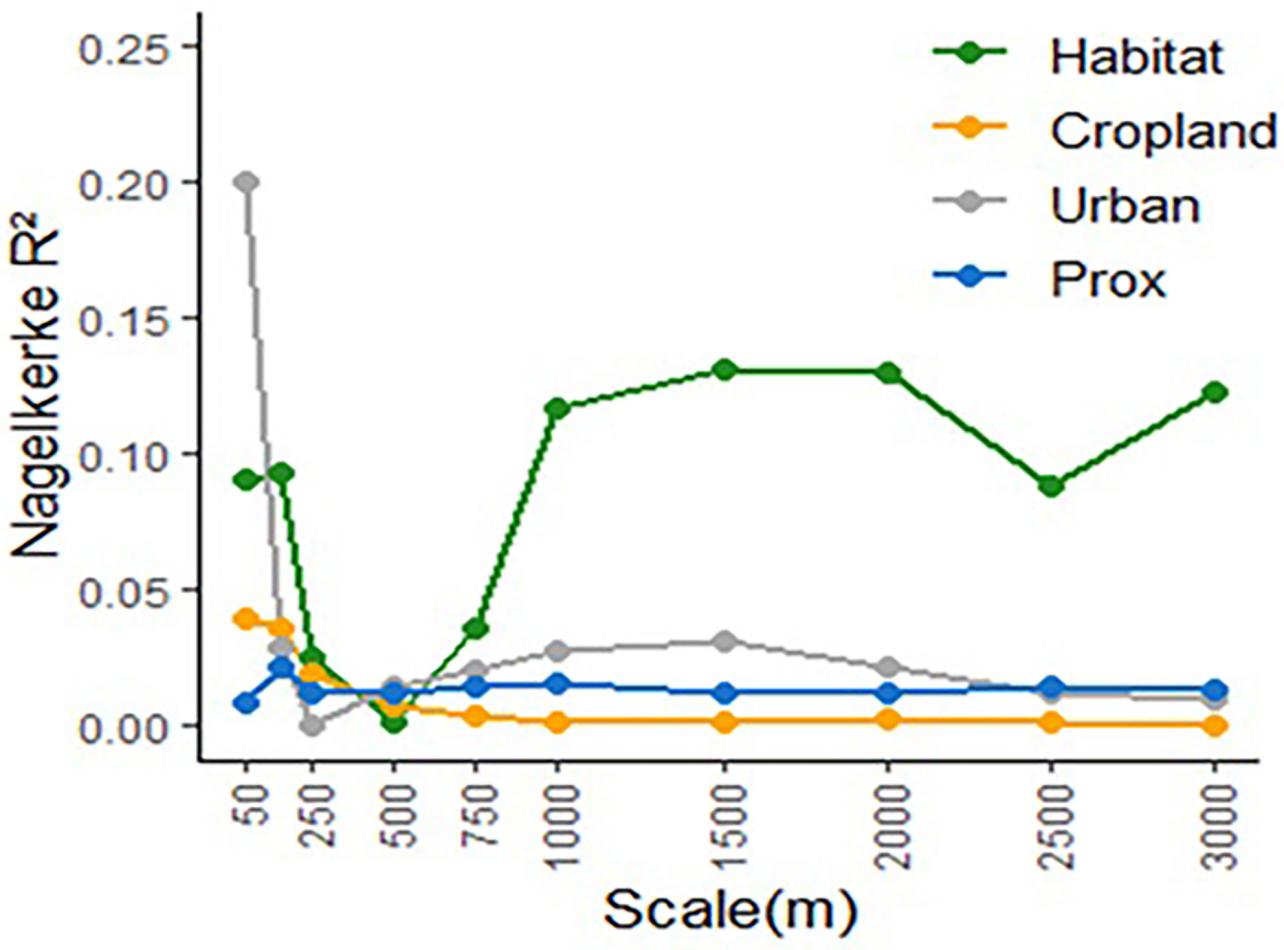
Scale of effect of scale‐dependent variables: Proportion of habitat (Habitat), proportion of cropland (Cropland), proportion of urban areas (Urban), and proximity index (Prox).

SoE of scale‐dependent variables is shown in Figure 4. Proportion of habitat had a large SoE, with its effect on density being stronger at 1.5 km around patches. On the contrary, the SoE's of proportion of cropland and proportion of urban areas, and of the scale‐dependent isolation measure prox, were small. The strongest effect of both proportion of cropland and proportion of urban areas were at 50 m scale, and for prox it was found at the 150 m scale.

### 3.2 Multiscale versus single scale

Results of the best selected model for the multi‐scale approach and for each single scale are presented in Table 1. Density was better explained by the multi‐scale approach, including landscape composition variables at their SoE's (N*R* ^2^ = .745, deviance change = 9.845), compared with the best model found at any single scale. With the single‐scale approach, density was better explained at the 500 m scale (N*R* ^2^ = .694, deviance change = 9.019).

**TABLE 1:** Best selected models explaining density of *L. viridis* with the multi‐scale approach and at each single scale.

Scale	Nagelkerke *R* ^2^	Deviance change	Area	Vegetation structure	Shape index	Distance to river	% habitat	% cropland	% urban
Multi‐scale	.7450	9.8458	+		−		−	−	−
50 m	.6330	8.0883	+	−	−			−	
150 m	.5740	7.1989	+		−			−	
250 m	.6460	8.2725	+			−	−	−	
500 m	.6940	9.0199	+			−			+
750 m	.6880	8.9277	+			−			+
1000 m	.6740	8.7131	+			−			+
1500 m	.6540	8.4093	+			−			+
2000 m	.6210	7.8958	+			−			+
2500 m	.5910	7.4457	+			−			+
3000 m	.5860	7.3744	+			−			+

*Note*: Each line represents the best selected model at the corresponding approach and scale. For each variable present in each selected model the direction of the effect is presented.

**FIGURE 5:**
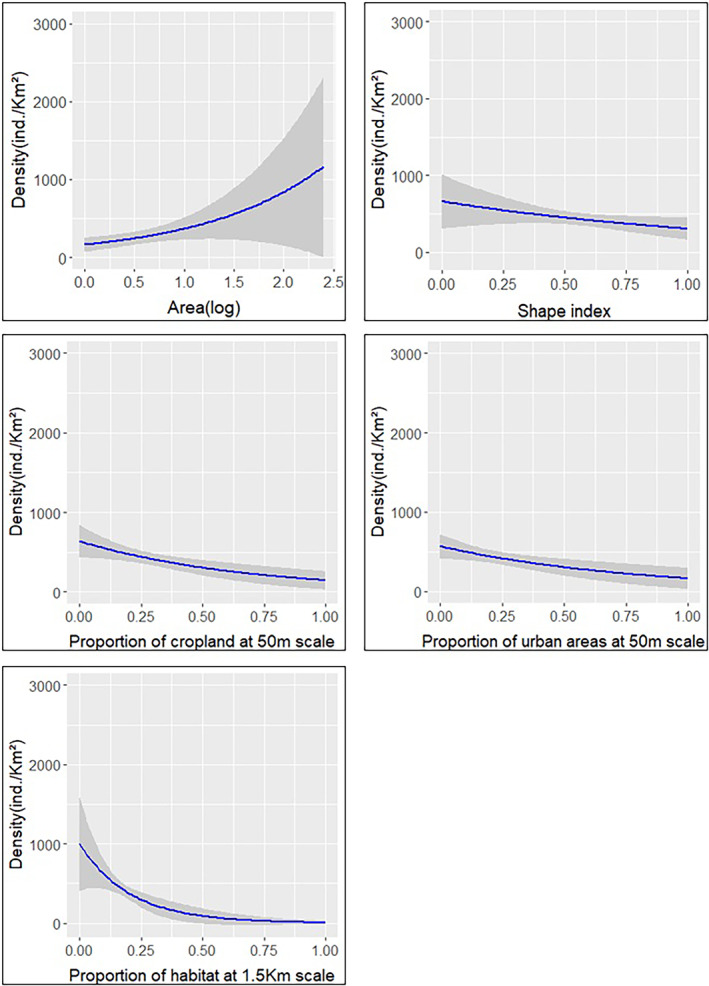
Effect of variables present in the best selected model in the multi‐scale approach on. The density of *L. viridis*. All predictor variables are plotted in their original values, except for Area, which is in logarithmic scale (log).

The variables explaining density in the best multi‐scale model included two patch geometry variables, area and shape index, and all landscape composition variables—proportion of habitat, cropland, and urban areas (Figure 5). Area had a positive effect on the population density of *L. viridis* (*β* = 0.824, SE = 0.194, *t‐*value = 4.239), while the effect of shape index (*β* = −0.768, SE = 0.475, *t*‐value = −1.615), and the three landscape composition variables was negative (Proportion of habitat: *β* = −4.835, SE = 1.676, *t*‐value = −2.884; Proportion of cropland: *β* = −1.481, SE = 0.528, *t*‐value = −2.801; Proportion of urban areas: *β* = −1.25, SE = 0.512, *t*‐value = −2.44).

**FIGURE 6:**
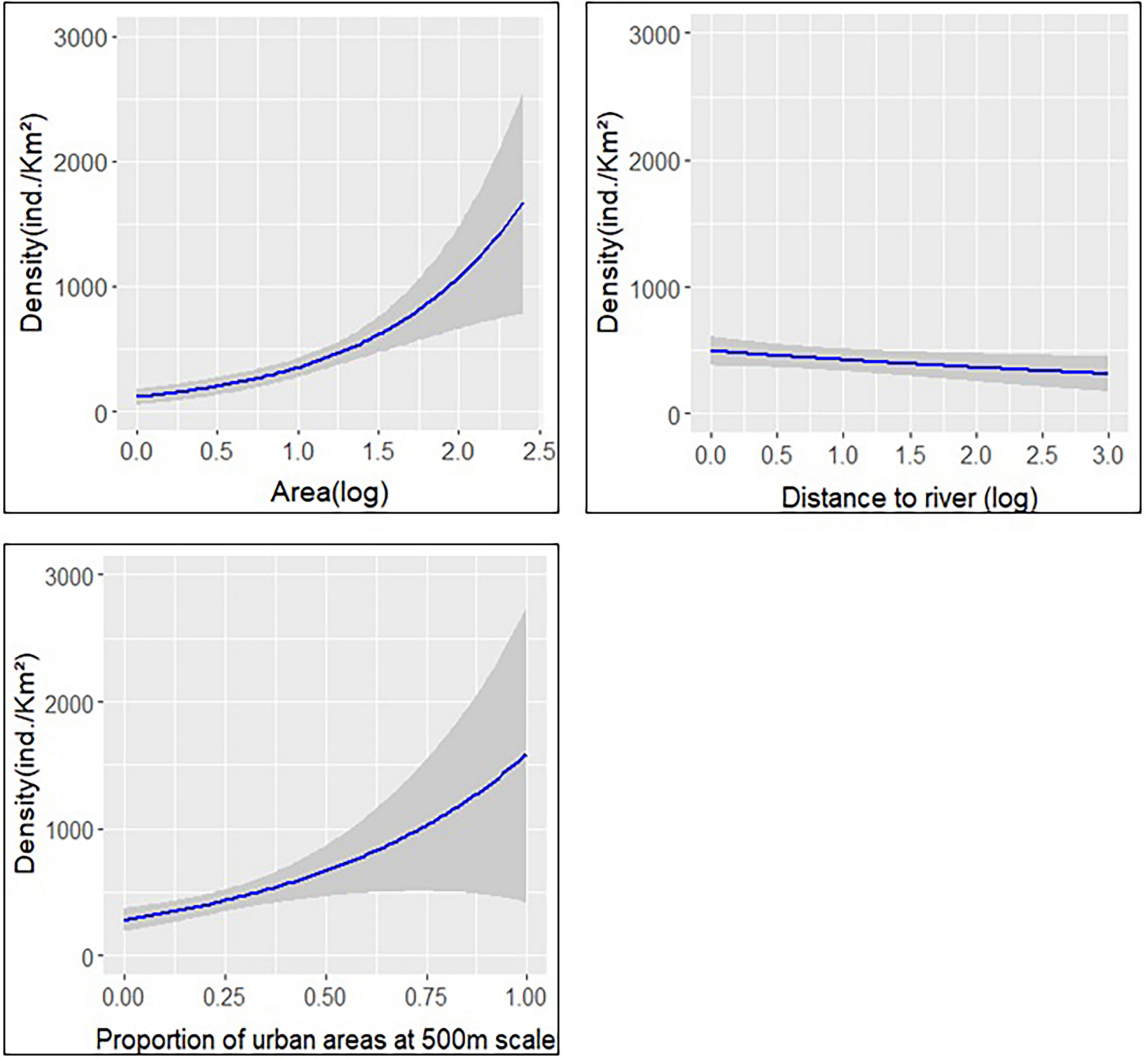
Effect of variables present in the best single‐scale model at 500 m on density of *L. viridis*. All predictor variables are plotted in original values, except for Area and Distance to river, which are in logarithmic scale (log).

The variables explaining density in the best single‐scale model at 500 m included area, distance to river, which is a variable representative of landscape configuration, and proportion of urban areas, which is a landscape composition predictor (Figure 6). Distance to river was found to have a negative effect on density (*β* = −0.152, SE = 0.087, *t*‐value = −1.739), while the effect of proportion of urban areas was positive (*β* = 1.73, SE = 0.489, *t*‐value = 3.532).

Area was the only predictor present across all selected single‐scale models, having a consistent positive effect on density. Other predictors present in selected single‐scale models show a clear spatial pattern regarding the range of scales at which they exert an effect on density. Shape index, vegetation structure, proportion of habitat, and proportion of cropland were present only at small scales, with all of them exerting a negative effect on density. By its side, distance to river was present only from the 250 m scale on, and its effect was consistently negative. Finally, proportion of urban areas was present only at medium and large scales and its effect was positive.

## 4 DISCUSSION

In this study, the effect of habitat loss on the population density of *L. viridis* in central Bulgaria was evaluated by combining a patch–landscape approach with the analysis of parameters at different spatial levels. Results do not support the first two hypotheses regarding positive effects of within‐patch habitat quality and no effect of patch size. On the contrary, the third hypothesis of no effect of isolation on population density was supported. Finally, the prediction of the fourth hypothesis, an effect at small spatial scales of individual landscape composition predictors and of the overall habitat loss process, was partially met. The SoE of both proportion of cropland and urban areas, was small (50 m), while the SoE of proportion of habitat was large (1.5 km). Also, the strongest effect of the overall habitat loss process was better described by the multi‐scale approach, with the best selected model including patch area, shape index and all landscape composition predictors at their SoE. These results partly contrast with those found for occupancy probability of the species in the same region, which was not affected by landscape composition parameters and was mainly defined by landscape configuration parameters and patch geometric characteristics (Prieto‐Ramírez et al., 2020).

The possible ecological processes underlying findings can be grouped into three aspects: habitat availability at the patch level, possible edge effects modulated by patch and landscape level predictors, and availability of habitat at the landscape level. Availability of habitat at the patch level is mainly represented by patch size, a predictor that had consistently a positive effect on population density across models, including single‐ and multi‐scale approaches. Positive effects of patch area on population density were also found in other reptile species (e.g., Rizkalla & Swihart, 2006; Shirk et al., 2014), and a meta‐analysis reported positive correlation of patch area with animal population densities in birds, insects, and mammals (Connor et al., 2000). Specially in landscapes with high habitat loss, large patches concentrate resources, like food, refuge, and mates, which in turn translate into positive reproduction and survival rates, and lower predation risk in comparison with small fragments. This can be the case in the studied system, where the total amount of habitat in the landscape was 11.2% (Prieto‐Ramírez et al., 2020).

By its side, possible negative edge effects on population density can be mediated at the patch level by the combined positive effect of patch area and negative effect of shape index (increases with patch irregularity). Patch interior increases with area and decreases with shape index, and therefore, population density of the species might depend mostly on available patch interior. At the landscape level, the SoE of proportion of cropland and of urban areas (50 m), at which their effect was negative, indicates an impact occurring at the direct vicinity of patches. Patch edges are hotter and drier than patch interior, given a higher exposure to surrounding land covers (Chen et al., 1999; Lehtinen et al., 2003), a phenomenon that can be more acute in scenarios of habitat loss (Arroyo‐Rodríguez et al., 2017; Laurance, 2004). Urban areas, for instance, have higher temperatures compared to natural or semi‐natural areas (Arnfield, 2003) and cropland could rise the exposure of patches to wind and water fluxes, thus triggering strong shifts in microclimatic conditions (Kapos et al., 1997; Saunders et al., 1991). Both could then affect the quality of patches in terms of lizard's microclimatic necessities for thermoregulation (Tuff et al., 2016) and developmental stability (Braña & Ji, 2000; Beasley et al., 2013; Lazić et al., 2013). This is especially important in subtropical and tropical regions, where sufficient cooler patch interior area is essential for reptiles to fulfill thermal physiological demands (Nowakowski et al., 2018; Todd & Andrews, 2008; Tuff et al., 2016). Additionally, cropland can affect density through negative edge effects on body condition due to exposure to pesticides, as found in *Podarcis bocagei* and *Podarcis muralis* (Amaral et al., 2012; Mingo et al., 2017) and to predators, like in populations of *Iberolacerta cyreni* (Amo et al., 2007). In the case of *L. viridis* in the studied region, Prieto‐Ramírez et al. (2020) concluded that the occupancy of the species depended on both enough patch interior and patch edge. This indicates that the effect of edge varies from population decline to population persistence, but also, that the importance of patch interior is consistent across processes.

Regarding availability of habitat at the landscape level, results suggest that its effect might be related with the spatial ecology of the species. Proportion of habitat had a negative effect in the best selected multi‐scale model, in which it was added at its SoE at 1.5 km. This SoE goes beyond the longest dispersal distance reported for *L. viridis* (1 km; Popescu et al., 2013), indicating that it is a suitable spatial scale to identify dispersal‐related processes. Moreover, Nemitz‐Kliemchen et al. (2020) found that the studied populations are not genetically differentiated, and therefore, might represent a metapopulation with considerable exchange of individuals. Thus, it can be expected that individuals seek to exploit resources in the available habitat outside patches and that this emigration from patches does decrease the density within patches. Furthermore, this effect seems to be counteracted by proportion of urban areas, whose effect on population density at medium to large scales (≥500 m) was positive, and which poses a barrier to the dispersal of *L. viridis*, resulting in the possible aggregation of individuals in isolated patches.

With respect to landscape configuration parameters, only distance to river seems to have a relevant impact on population density. Although this predictor was not included in the best multi‐scale model, it was present in all selected single‐scale models from scale 250 m on, including the best selected single‐scale model at 500 m, having a negative effect on population density. Prieto‐Ramírez et al. (2020) found negative effects of distance to river on occupancy probability and suggested riparian vegetation to act as a corridor. Therefore, as in the case of percentage of habitat, this parameter of landscape configuration might also promote dispersal of individuals and reduce their density within patches. On the other hand, any measure of patch isolation was found to have an effect on population density. This finding is in accordance with the HAH (Fahrig, 2013), which states that in landscapes with high levels of habitat loss, habitat amount as composition‐based parameter reflecting isolation, affects species distribution much more strongly than distance, configuration‐based parameters of isolation (Martin & Fahrig, 2012).

Similarly, any of the two evaluated within‐patch habitat quality parameters, solar radiation, or vegetation structure, were included in the best selected multi‐scale or single‐scale (500 m) models. The occupancy probabilities of the species in this region were also found to have a lower dependency on habitat quality compared with the periphery (Prieto‐Ramírez et al., 2020). This might be related to the fact that *L. viridis* is a generalist species, having a bigger realized niche at the core, where the studied region is located, compared to populations in the periphery of its distribution range (Prieto‐Ramírez et al., 2018). Habitat generalization is positively related with capacity to thrive in modified landscapes (Ye et al., 2013), and in reptile communities, low dependency on habitat quality has been found to positively correlate with niche breadth and proximity to the core of the distribution range of species (Rizkalla & Swihart, 2006; Swihart et al., 2006).

Population density and patch occupancy reflect important ecological processes of wild animal populations in modified landscapes, namely population decline and persistence. However, information on occupancy and density or abundance is available only for very few species, and in the reptile literature, only some authors have integrated both approaches in the same study region (e.g., Dibner et al., 2017; Lizana‐Ciudad et al., 2021). This might be due to the challenges of fulfilling the data‐gathering requirements of both types of parameters in a single survey. Occupancy surveys are usually suggested to be uniform, applying the same sampling effort in each patch, in order to not affect detection probability (Cristescu et al., 2019; Krishna et al., 2008). On the other hand, abundance and density studies are suggested to have a proportional sampling effort, in which the entire area of each patch (which is usually variable) is surveyed (Nufio et al., 2009). In this study, data to estimate population density were gathered during the same field season in which occupancy data was collected (Prieto‐Ramírez et al., 2020), by applying a semi‐uniform survey design. All transects within a patch summed up the same total length, and therefore, sampling effort across patches was standardized. However, the number and length of single transects, in which that total length was partitioned within each patch, was proportional to the number and area of habitat types within each single patch. Thus, the survey was “proportional” with respect to how the heterogeneity of each patch was reflected. This is a robust combination of survey design types, solving mismatches between occupancy and abundance data gathering methods.

Concerning the necessary conservation measures for *L. viridis* in the studied region, Prieto‐Ramírez et al. (2020) highlighted the importance of protecting and restoring riparian vegetation, which might be an important corridor connecting populations, to increase the occupancy probability of remnant patches. In addition to this recommendation, early conservation measures to avoid the decline of still extant populations of *L. viridis* should include ensuring enough patch interior area, restoring habitat at small scales (~50 m), at which cropland and urban areas are exerting strong negative pressure, and protecting and restoring habitat at large scales (~1.5 km), which cover the species' maximum dispersal distance and at which connectivity can be much more strengthened.

Understanding the response of wild animal populations to habitat loss at different stages of the population extinction process, and at different spatial levels, is of vital importance to identify the best possible conservation measures. Hence, the present study shows how important it is to complement studies evaluating the effects of habitat loss on occupancy with those assessing effects on density, applying a spatial multi‐level approach. This can lead to much more effective conservation plans aimed at protecting endangered animal species.

## AUTHOR CONTRIBUTIONS

**Ana María Prieto‐Ramírez:** Conceptualization (equal); data curation (equal); formal analysis (equal); funding acquisition (equal); investigation (equal); methodology (equal); project administration (equal); resources (equal); software (equal); supervision (equal); validation (equal); visualization (equal); writing – original draft (equal); writing – review and editing (equal).

## CONFLICT OF INTEREST STATEMENT

None declared.

## Data Availability

Data are available in the following Dryad repository: https://datadryad.org/stash/share/OJzKAdjy7du7gpzuTQYSjN8AVNENVvdavij2viamf2Q.

## APPENDIX 1

### 1.1

See Table A1.

**TABLE A1:** Results of models tested for detection probability function.

Model	Delta AIC	AIC	%CV	K‐S test estimate	K‐S test *p*‐value
MCDS hazard.rate, simpl.polyn, var.emp	0.0	1165.35	4.66	0.1246	.0720
MCDS hazard.rate, simpl.polyn, var.poisson	0.0	1165.35	5.33	0.1246	.0720
**CDS uniform, hermit, var.emp**	1.92	1167.27	4.02	0.1199	.0924
CDS uniform, hermit, var.poisson	1.92	1167.27	4.02	0.1199	.0924
CDS uniform, simpl.polyn, var.emp	1.92	1167.27	4.02	0.1199	.0924
CDS uniform, simpl.polyn, var.poisson	1.92	1167.27	4.02	0.1199	.0924
CDS half.norm, simpl.polyn, var.emp	2.48	1167.83	9.62	0.1469	.0198
CDS half.norm, simpl.polyn, var.poisson	2.48	1167.83	9.62	0.1469	.0198
MCDS half.norm, simpl.polyn, var.poisson	4.0	1169.35	5.99	0.1479	.0185
MCDS half.norm, simpl.polyn, var.emp	4.0	1169.35	5.99	0.1479	.0185

*Note*: Conventional distance sampling (CDS) and multivariate conventional distance sampling (MCDS) models were evaluated. The fit of three functions was tested: uniform key (uniform), half‐normal key (half.norm) and hazard rate. Also three types of adjusted terms cosine, Hermit polynomial (hermit) and simple polynomial (simpl.polyn). Finally, two methods for calculating encounter rate variance were also tested, empirical (var.emp) and assuming distribution of observations as Poisson (var.poisson) were tested. Results show only models that converged without warnings.

## APPENDIX 2

### LOCATION OF THE STUDIED POPULATIONS AND THEIR DENSITY ESTIMATION

**Table:**

Latitud	Longitud	Density estimation (ind./km^2^)	Lower CI 95%	Upper CI 95%	Patch area (km^2^)
42.1424251554	24.7005365441	1614.3	1490.8	1748.1	1.6528
42.1558369207	24.751854855	115.31	106.48	124.87	0.0069
42.1530052654	24.7341106897	576.54	532.42	624.33	0.0122
42.1638705559	24.7627120874	230.62	212.97	249.73	0.1051
42.159716059	24.775973976	345.93	319.45	374.6	0.0421
42.1622382296	24.7972366471	461.24	425.93	499.46	0.3978
42.1559844181	24.7638739964	1953.5	1804	2115.4	0.1027
42.1486304047	24.7074005835	381.71	352.5	413.35	0.3379
42.1528700357	24.7064799434	115.31	106.48	124.87	0.0308
42.157272404	24.717881851	115.31	106.48	124.87	0.0363
42.164088255	24.77076136	230.62	212.97	249.73	0.2494
42.1904096804	24.7691450069	230.62	212.97	249.73	0.0776
42.1950769481	24.7754071989	345.93	319.45	374.6	0.0532
42.1986480095	24.7590213774	230.62	212.97	249.73	0.0812
42.1248176838	24.8669810476	638.63	589.75	691.57	0.4226
42.15098957	24.882848019	461.24	425.93	499.46	0.7157
42.151966833	24.816918005	230.62	212.97	249.73	0.2823
42.1246113013	24.8685782244	807.16	745.38	874.06	0.4582
42.2122746411	24.8676029012	345.93	319.45	374.6	0.0807
42.2285590342	24.8579243368	1284.9	1186.5	1391.4	2.0205
42.2245713336	24.8831351044	732.55	676.48	793.27	1.326
42.2060170746	24.8986887584	230.62	212.97	249.73	0.1771
42.199659108	24.8893898943	691.85	638.9	749.2	0.1873
42.226213075	24.848176836	509.79	470.77	552.04	0.4341
